# Degree of enhancement: A theoretical and formal definition

**DOI:** 10.3389/fpsyg.2022.1072423

**Published:** 2023-01-09

**Authors:** Federico Cassioli, Michela Balconi

**Affiliations:** ^1^International research center for Cognitive Applied Neuroscience (IrcCAN), Università del Sacro Cuore, Milan, Italy; ^2^Research Unit in Affective and Social Neuroscience, Department of Psychology, Università Cattolica del Sacro Cuore, Milan, Italy

**Keywords:** human enhancement, degree of enhancement, NBCI, conceptual issues, theoretical

## 1. Definitions of human enhancement: A critical analysis

Enhancement extends our capacities and its applications are value-charged, never neutral. The purposes are—but are not limited to—increasing productivity, creativity, lifespan, and fertility, improving body and mind, and choosing peculiar genetic characteristics (Menuz et al., 2013). Toward enhancement, there are permissive (e.g., Earp, 2019), restrictive (e.g., Buttrey et al., 2022), and conservative (e.g., Fukuyama, 2003; Cohen, 2012) positions. In the continuum, conservationism stands for the complete preservation of human nature (Schermer, 2008), while transhumanism argues that the application of radical forms of enhancers is a natural evolutionary consequence and thus should be actively pursued (Lyreskog and McKeown, 2022). Some authors bisect enhancement into “traditional" and “modern” (Kudlek, 2022). All mechanisms (e.g., clothing, writing, language) that have led to the current notion of *Homo sapiens* and boosted humans beyond the *normal* range can be viewed as an enhancement (Caplan, 2009). Modern enhancement instead solely refers to the applied convergence of nanotechnology, biotechnology, informational technology, and cognitive sciences (NBCIs).

In the contemporary debate, as nicely worded by Juengst and Moseley (2019, p. 12), “the hidden assumption is that the moral problems raised by enhancement intensify as the enhanced move away from the human norm.” The moral philosophical dispute further lies upon the pre-assumptions regarding the enhancement purposes or the expected outcomes.

Since enhancement raises moral problems, drawing lines, a threshold, to define the misuse of enhancement practices, seems a priority. We argue that to draw a line, you need a plane. Moreover, we partially agree only with a clear-cut dichotomy between traditional and modern enhancers. This distinction appears imprecise and depends on the sociocultural values prevailing in a given epoch (Menuz et al., 2013). We further note that, as society progresses, the *degree of enhancement* (DoE) of an entity toward an individual might relatively vary, as a function of social-technological advancements. By “degree,” we here refer to it as the extent of the enhancement effects on an individual in all their facets (e.g., social, physical, psychological, genetic). Specifically, we affirm that, due to adaption, DoE is generally subject to variation and strictly connected to what we consider normality and wellbeing. Another issue we found, refers to the absence of a DoE's theoretical definition. The literature shows uncertainty regarding the concept of human enhancement, and pure vagueness for the DoE (Agar, 2013). Without a proper formalization, how can the nature of two enhancers be established? How to pick a certain ethical position to draw public policy and professional practice?

Thus, the scope of this work is to critically discuss the definition of enhancement, from which we will derive a DoE conceptualization and, ultimately, a revised characterization of enhancement. The idea of DoE is not innovative. Scholars had indirectly mentioned radical enhancers (vs. moderate) before (Rueda, 2022), or “degrees of enhancement” (e.g., Brownsword, 2009), and affirmed that “human enhancement is a good thing, but one that it's possible to have too much of” (Agar, 2013, p. 1). Although, to the author's knowledge, no proper theoretical conceptualization has ever been developed. According to the President's Council on Bioethics, NBICs' applications furnish improvements beyond the species-typical level or statistically-normal range of functioning (Kass et al., 2003). Linking the definition of enhancement to typical normal functioning or complex constructs (i.e., wellbeing or intelligence) is surely troublesome, but might be the most formally accurate solution.

Stricto sensu, enhancing means (i.) intensify, increase, or further improve the quality, value, or extent of something (McKean, 2005). A biotechnological definition sees (ii.) human enhancement as those interventions that improve human capacities, performances, disposition, and wellbeing, beyond the scope of therapy (Giubilini and Sanyal, 2016). As set out above, (iii.) enhancement is what exceeds the treatment, and goes beyond “species-typical normal function” and “standard capacities” (Daniels, 2000; Missa, 2009). The prescription of synthetic erythropoietin (EPO) exemplifies this distinction: EPO is labeled as therapeutic for kidney diseases and, as enhancement, if used to higher the human *normal* levels of red blood cells (Ansah, 2022). In addition, neuroenhancement (iv.) is seen as the increase of cognitive functioning through the use of different neuroscientific technologies that operate on the nervous system, altering certain properties in a specific cognitive task (Balconi and Crivelli, 2020). Other perspectives had defined it (v.) as any change in the biology or psychology which increases the chances of leading a good life in a given context (Savulescu et al., 2014). Lastly, a definition that appears valuable comes from Menuz et al. (2013). They propose to consider whether the outcome of a given technological intervention can be described as enhancement or not (vi.), by employing the individual's determination associated with her/his *personal optimum state* (POS, Bates, 2022).

While we agree that the contextual (historically, socially, and techno-scientifically determined) and probability (“chance”) factors are involved, the formalization in *v*. adds an extra layer of subjectivity, for example, based on the personal definition of what is a “good life.” Moreover, the POS-based approach alone is not conceptually self-standing, since it allows a too vast degree of subjectivity, but we agree that DoE is perception-determined as well.

## 2. Degree of human enhancement: A conceptual formalization

Given an enhancement entity *e*, we aim at estimating its DoE.

From the definitions (i.e., definitions i, ii., iii., iv.), we learned that *e* provokes a change (Δ), which *increases* certain properties *n*_1,2…*n*_ (Δ >0). The changes respond specifically to and are manifested *via* alterations of a subject's mental or physical characteristic. For example, to examine the effect of technology-enhanced language support on vocabulary proficiency, the difference between pre-and post-scores of vocabulary tests was considered (e.g., Gay, 2022), as in Equation 1.


(1) $$\begin{array}{l} DoE={\text{n}1}_{e}-{\text{n}1}_{ne}={\text{Δ}}_{n1} \end{array}$$

Given the concept's multidimensionality, the significant measurable dimensions (*n*) for every enhancer are most likely many and domain-specific. Hence, DoE logically has to be directly proportional to each of the differences between the enhanced (*nn*_*e*_) and the non-enhanced (*nn*_*ne*_) measures. Moreover, as noted by Agar (2013), a negative enhancer, sub-dimensionally speaking, is not an oxymoron. Ritalin may enhance concentration but reduce creativity.

We then operationalize this in Equation 2 as the summation of each Δnn, in percentage (by dividing Δnn for the measured dimension without enhancer, nn_ne_). Since the proposed dimensions could present different loads, weights were added (*k*_n_, with ∑ *k*_n_=1). This allows focusing on specific variables, by setting *k*_n_ to zero. (e.g., a pharmaceutical company, in a first step, might have interest in measuring the *e*'s effect on concentration only).


(2) $$\begin{array}{l} DoE=\sum \left(k_{1}\frac{{\text{Δ}}_{n1}}{{n1}_{ne}}\right);\left(k_{2}\frac{{\text{Δ}}_{n2}}{{n2}_{ne}}\right);\ldots \left(k_{n}\frac{{\text{Δ}}_{nn}}{nn_{ne}}\right) \end{array}$$

Let us now furnish an example. Neurocognitive (e.g., faster response times in a Stroop test), doping, or physiological enhancements (e.g., faster race) are examples where the enhancers' effects are very *instrumentally valuable*. A cognitive enhancement of greater magnitude enables the solution of more difficult problems. In this case, we could state that *e*'s impact is very time-dependent (*k*_t_ tends to 1). Thus, the difference in time (Δ*t*) between the required time to perform a task and the required time to perform the task with *e*, can be taken into consideration. Logically, Δ*t* must be directly proportional to the *e*'s DoE. If reliability is assumed, by considering the time dimension, a high degree of objectivity is built up. Since an enhancer could just provoke easiness in the execution (if we concentrate on the intrinsic value of our capacities), time alone is necessary but non-sufficient.

Furthermore, Equation (2) is constituted of individual-dependent factors: the personal characteristics of the chosen person only influence the degree of enhancement.

Earlier in the work, we concluded that DoE is inversely proportional to the human average capacity in the chosen task without *e*. We further added that enhancement is also a function of general socio-technological advancements. Some centuries ago, the use of a calculator could have been considered a great cognitive enhancement, but today we should not. We can deduce that as society progresses, DoE can be (not necessarily) subject to decay. Conversely, by using a certain enhancer, previous methods might be weakened (the fishing rode might have reduced the ability of fishing by hand). We determined that evolutionary and environmental factors intervene, and a link to the species-typical level or statistically-normal range of human functioning exists. As noted by Menuz et al. (2013) it is arduous to determine a “normal functioning” for specific traits or cultural identities (Chadwick and OConnor, 2013).

For these many listed reasons, we propose to consider *p*_1_, as the probability of a subject directly taken from the *population of interest*, to perform *n*1_e_ with the available resources they have (i.e., without *e*). Logically, *p*, appears to be inversely proportional to *DoE*. We derived Equation 3.


(3) $$\begin{array}{l} Instrumental\;DoE=\sum \left(k_{1}\frac{{\text{Δ}}_{n1}}{{n1}_{{ne}^{*}}p_{1}}\right);\left(k_{2}\frac{{\text{Δ}}_{n2}}{{n2}_{{ne}^{*}}p_{2}}\right);\\ \;\ldots \left(k_{n}\frac{{\text{Δ}}_{nn}}{nn_{{ne}^{*}}p_{n}}\right) \end{array}$$

In Equation 3, we successfully described DoE for its instrumental value. For certain enhancers—or better, certain scopes—this can be already seen as a comprehensive description. Although, as noted by philosopher MacIntyre (2016), internal goods have been systematically unrecognized, in favor of instrumental-oriented definitions. The attributed value of the experience needs to be heeded.

It is 1997, and IBM's Deep Blue beats Garri Kasparov. Despite this historical turning point, World Chess championships have not ceased to exist. We do genuinely engage with Kasparov, not with Deep Blue. The grandmaster plays in a way we value and experience by proxy. Beyond a certain DoE threshold, we tend to be disconnected and attribute smaller intrinsic value to the enhanced outcome. If “drastic” enhancers are involved, humans are not interested in athletes running a sub-2-h marathon. We are psychologically detached. Thus, the value of the performance is a function of our evaluative connection, not with others, but with our future selves and performances. In definition vi., the authors suggested considering the individual's determination. We propose to assume that the attributed value, strictly related to personal engagement, interest, and POS, reflects the overall intrinsic value factor, as the difference between the subjective evaluations of the enhanced (*exp*_*e*_) and non-enhanced (*exp*_*ne*_) experiences, as described in Equation 4. Here we wish to clarify that internal goods are not necessarily more important than instrumental value, and each of the two components could be discarded for specific purposes.


(4) $$\begin{array}{l} Experience\;Evaluation={\text{exp}}_{e}-{\text{exp}}_{ne}={\text{Δ}}_{\text{exp}} \end{array}$$

The ending results combining Equations 3 and 4 is 5 and represents DoE composed of the individual's perceived experience evaluation (Δ_exp_, representative of the intrinsic value and the POS) and the individual's instrumental value [∑_(*k*_*n*_(Δ*nn*_/*nn*_*ne*_))], as a function of the considered *n* dimensions and the probability *p*_n_ to achieve *nn*_*e*_, for each dimension, in the population of interest.


(5) $$\begin{array}{r} Total\;DoE=\left[1+{\text{Δ}}_{\exp}\right][1+\sum \left(k_{1}\frac{{\text{Δ}}_{n1}}{n1_{ne^{*}}p_{1}}\right);\\ \;(k_{2}\frac{{\text{Δ}}_{n2}}{n2_{ne^{*}}p_{2}});...\left(k_{n}\frac{{\text{Δ}}_{nn}}{nn_{ne^{*}}p_{n}}\right)] \end{array}$$

The Equation 5 mirrors the following definition we now propose. Human enhancement is a complex notion that manifests itself *via* alterations in the personal subjective experience and/or in certain instrumentally-charged properties, each in relation to the norm of the population of interest, with a certain weight on the ultimate enhancing effect.

## 3. Conclusions: Doe' strengths and limits

We will now uncover the strengths and limits of the work we carried out. We believe that DoE represents a novelty in the literature because it encapsulates currently available definitions of enhancement, in all their features.

It allows the measurement of human enhancement with universal applicability, considers both its subjective and instrumental value, and admits the existence of negative enhancing sub-effects. Moreover, this theoretical formulation allows cross and longitudinal comparisons (inter-DoE), because places the measurement in a socio-technological environment (e.g., the Italian population in the early 1990s vs. 2000s) and for a certain population of interest (e.g., healthy vs. neglect population). It could be applied in ethically-charged issues or to evaluate novel technologies, such as the past use of body polyurethane suits in swimmers (Foster et al., 2012) or implantable brain devices (Ireni-Saban and Sherman, 2021). Lastly, the weights grant to deal with factors that are expected to differently load on DoE (e.g., a cognitive task measured according to the method of subtraction, *k*'s reaction times tends to 1) and respond to specific research needs (compute instrumental value only, or vice versa).

Regarding the limits, we first would like to state that DoE might be theoretically meaningful but empirically fragile. We further express doubts about the actual applicability of DoE. Evidence needs to be gathered for validation and adjustment purposes.

For sure, Δ_nn_ should strictly reflect the actual enhancement. Careful attention to the considered dimensions (and their interdependency), to proper measurement (e.g., IQ scores showed low statistics reliability), to isolate the confounding variables, and in the selection of the target population, should be put. Lastly, it should be noted that, being historically dependent, if a certain enhancer becomes the norm, DoE incorporates this change *viap*_n_ (e.g., if a population of athletes commonly uses doping substances, their doped performance is used as a benchmark).

In the future, given the ubiquity of enhancement applications, numerous transdomain tests on our theoretic proposal should be carried out.

## Author contributions

FC conceived and developed the theoretical framework of the manuscript and wrote a first draft. FC and MB commented and contributed to the final version of the manuscript. All authors contributed to the article and approved the submitted version.
